# Anatomical Landmarks for Transverse-Sigmoid Sinus Junction: A Cadaveric Study

**DOI:** 10.7759/cureus.59278

**Published:** 2024-04-29

**Authors:** Jonas Jurgaitis, Šarūnas Jukna, Gunaras Terbetas

**Affiliations:** 1 Neurosurgery, Faculty of Medicine, Vilnius University, Vilnius, LTU; 2 Neurosurgery, VšĮ Republican Vilnius University Hospital, Vilnius, LTU

**Keywords:** junction of the transverse and sigmoid sinus, digastric groove, mastoid notch, sigmoid sinus, transverse sinus, retrosigmoid approach

## Abstract

Background and objective: Accurately identifying and avoiding crucial anatomical structures within the posterior cranial fossa using superficial landmarks is essential for reducing surgical complications. Our study focuses on the top of the mastoid notch (TMN) as an external landmark of the cranium, aiming to assist in the strategic placement of the initial burr hole. In this study, we present a method for predicting the path of the transverse sinus (TS) and explore the relationship between the junction of the transverse-sigmoid sinus and the TMN.

Methods: Following anatomical dissections of the brain in cadaveric specimens, we conducted intracranial drilling from the inside surface of the cranium on 10 adult skulls (20 sides). A coordinate system was established on the posterolateral surface of the skull to assist the analysis. Using a self-leveling laser level, we set up a horizontal Frankfurt line (X-axis) and identified a vertical perpendicular line passing through the TMN to serve as the Y-axis. To identify the course of the TS, we divided the segment between the two inferomedial points into six equidistant points along the Frankfurt line.

Results: No significant difference was observed between the inferomedial points of the transverse-sigmoid sinus junction (TSSJ) on the left and right sides. The inferomedial point was positioned at a median of 6.6 mm (Q1: 3.7 mm, Q3: 9.4 mm) dorsally and at a median of 19.2 mm (Q1: 16.1 mm, Q3: 23.2 mm) cranially from the TMN. The upper edge of the TS was located at distances of 6.4 mm (5.7; 12.7), 10.3 mm (8.8; 12.3), and 13.8 mm (11.9; 16.3) on the right, and 4.9 mm (4.1; 7.9), 8.6 mm (7.6; 13.0), and 12.8 mm (11.7; 17.5) on the left side from the Frankfurt horizontal plane at the ¼, ½, and ¾ line points, respectively. The bottom edge was positioned at distances of 0.6 mm (-2.7; 2.0), 2.1 mm (-0.8; 3.8), and 4.8 mm (2.4; 6.7) on the right, and 1.1 mm (-3.4; 2.4), 2.0 mm (0.2; 4.8), and 3.9 mm (3.7; 5.3) on the left from these respective points. The upper edge of the right TS was found to be statistically more distant from the Frankfurt horizontal plane at the ¼ line point (p-value = 0.027) compared to that on the left side. The confluence of the sinus center was identified as having a median distance of 7.8 mm (4.5; 8.3) and an inferior point of 1.5 mm (0.1; 3.0) cranially to the inion. In all examined bodies (n = 10), the confluens sinuum was consistently 4.7 mm (3.3; 5.6) to the right in relation to the inion. Notably, the median of the right transverse sinus diameter (median = 9.3 mm) was found to be significantly larger than that of the left transverse sinus (median = 7.0), with a statistically significant p-value of 0.048.

Conclusions: The literature regarding the external identification of the TSSJ and the course of the TS varies. In our efforts to provide a description, we have utilized the TMN as a reliable landmark for locating the TSSJ. To delineate the trajectory of the TS after its exit from the confluence of sinuses, we employed a Frankfurt horizontal plane to the inion. These findings may assist surgeons by using external skull landmarks to identify intracranial structures within the posterior fossa, particularly when image guidance devices are not available or to complement a neuronavigational system.

## Introduction

The part of the cranial cavity located between the foramen magnum and the tentorium cerebelli, known as the posterior cranial fossa, represents a common ground for neurosurgeons. Suboccipital craniotomies are among the most frequently used approaches for addressing pathology within the posterior part of the cranial cavity. These procedures are commonly utilized for conditions such as tumors in the pontocerebellar angle, cerebellar metastases, hemorrhagic strokes, trigeminal neuralgia, and other related disorders.

The posterior cranial fossa is a distinct part of the cranium primarily because of its venous sinuses. The transverse sinus (TS) is often invaded by tumors, with tentorial meningiomas being the most common cause. Meningiomas that affect the sigmoid sinus are rare, accounting for only 10% of all meningiomas [1]. In addition, dural arteriovenous fistulas most frequently occur at the transverse-sigmoid sinus junction (TSSJ) [2]. Traumatic injury affecting the TS is the second most frequent type of venous sinus injury, accounting for 18% to 24% of all cases. Moreover, injuries involving the TS have been reported to cause a mortality rate of 29% and they frequently occur alongside the sigmoid sinus or at the TSSJ [3]. As a result, it is crucial for a surgeon to have a comprehensive understanding of the area's topography, particularly to prevent iatrogenic damage of the venous sinuses. Especially, while dealing with tumor removal, craniotomy, or trepanation.

The initial burr hole (known as the keyhole) placement is a critical aspect of the standard posterior fossa cranial base approach, as it helps to minimize bone loss, ensuring the best exposition of important anatomical structures. Secondly, injury to the underlying venous sinuses can result in intraoperative complications and postoperative neurological issues. Neurosurgery still relies on anatomical localization, especially in the posterior fossa where neuronavigation is less reliable [4]. There have been several efforts to pinpoint the anatomical markers that represent the TSSJ. Superficial reference points of the cranium, including the asterion, the superior nuchal line, the line connecting the inion, and the root of the zygomatic arch are employed to identify significant intracranial structures [5]. To summarize, the approaches have the following drawbacks: firstly, there are instances where the skull sutures cannot be clearly distinguished, resulting in ineffective localization of the TSSJ. Secondly, conventional reference points like the asterion lack the necessary precision to accurately locate the TSSJ [6,7]. Lastly, utilizing image-guided surgical planning is costly and time-consuming.

The mastoid process has a distinct groove called the mastoid notch, which is located on its medial side and easily recognizable. Due to its proximity to the TSSJ, the top of the mastoid notch (TMN) can serve as a useful reference point for identifying the anterosuperior (AS) and inferomedial (IM) points of the TSSJ [8-10]. The TMN, also called the digastric point, is defined as the most superomedial point of the notch and is always regarded as the lateral limit of the retrosigmoid craniotomy.

The objective of this study was to identify variations of venous sinuses in the posterior cranial fossa and examine their relationship with external skull bony landmarks in a formalin-fixed cadaveric cohort.

## Materials and methods

The study involved 10 formalin-fixed adult cadavers of Lithuanian origin (four males and six females) that were obtained from the Anatomy, Histology, and Anthropology Department of Vilnius University in Lithuania. Before their passing, all the participants had provided informed consent. None of the individuals displayed any signs of structural abnormalities affecting cranial or cerebral integrity, such as skull fractures, craniosynostosis, previous craniotomy, or cerebrovenous disorders.

**Figure 1:**
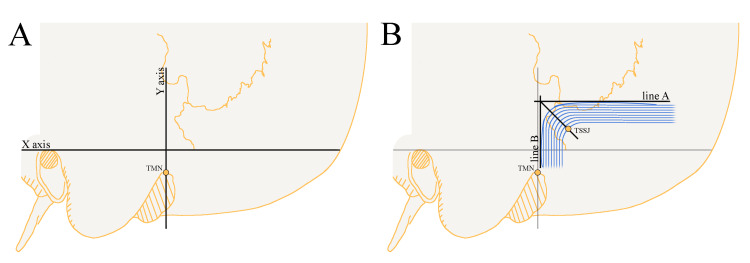
Definition of the TSSJ used in this study. (A) The illustration schematically depicts a Frankfurt horizontal plane extending to the inion (X-axis) and a perpendicular line passing through the TMN (Y-axis). These lines were used to determine the coordinates of the IM point of the TSSJ. (B) The illustration shows both the intracranial transverse-sigmoid sinus junction and landmarks situated outside the cranial region. The TSSJ in this study was defined following the description by Teranishi et al. [11]. Using a laser level, a transverse line (line A) was drawn tangent to the rostral curve of the transverse sinus, and a sigmoid line (line B) was drawn tangent laterally to the curve of the sigmoid sinus. The TSSJ was defined as the point of intersection between the inferomedial point of the sinus and a line dividing the angle formed by lines A and B into two equal parts. TSSJ: transverse-sigmoid sinus junction; TMN: top of the mastoid notch; IM: inferomedial. Image Credits: Jonas Jurgaitis

After the soft tissues of the head were separated from the cranium, using a self-leveling laser level, a Frankfurt horizontal plane to the inion and a perpendicular line passing through the TMN were identified to serve as the X- and Y-axes, respectively, to determine the coordinates of the IM point of the TSSJ. Positive values were assigned to the posterior side on the X-axis, while negative values represented the anterior side. The digastric point or TMN represents the origin (point 0) of the Y-axis. On the Y-axis, the superior side was positive, and the inferior side was negative (Figure 1A). The TMN was used as our reference point in establishing coordinates, as it is one of the most easily felt or seen bony landmarks in the surgical area for retrosigmoid craniotomy, whether before or after making an incision in the skin. After the removal of the posterior cranial vault, together with the mastoid processes and the sinuses of the posterior cranial fossa, the IM point was determined with a laser level.

The TSSJ has multiple definitions, and only a handful of reports provide a precise one. As a result, we used a single point to define the TSSJ. Specifically, with the help of a laser level, a transverse line (line A) was drawn as a tangent rostrally to the curve of the transverse sinus, and a sigmoid line (line B) was drawn as a tangent laterally to the curve of the sigmoid sinus. The intersection between the inferomedial point of the sinus and the line that divides the drawn angle (between lines A and B) into two equivalent parts was defined as the TSSJ. Just as it was outlined by Teranishi et al. [11] (Figure 1B).

To accurately describe the position of the inferomedial point of the TSSJ, we employed a designated coordinate system. The TMN was established as the origin (point 0) for the ordinate axis (Y). The X-axis, representing the Frankfurt horizontal plane extending to the inion, was utilized to determine X-values.

**Figure 2:**
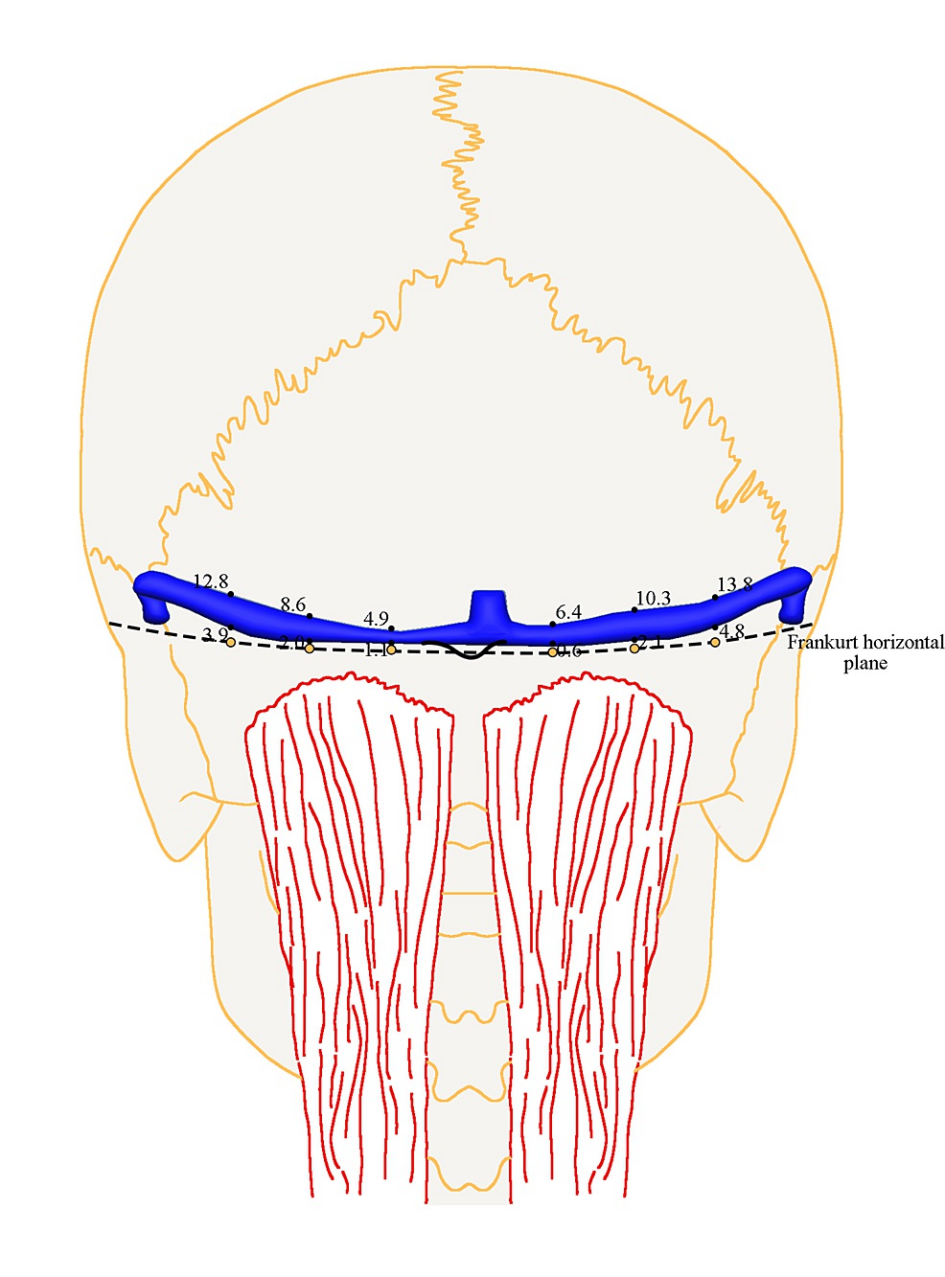
Schematic representation of measurements in mm based on reference points. A Frankfurt horizontal plane (FHP) extending to the inion was marked using a laser level. The distance along this line was measured from the inferomedial point of the transverse-sigmoid sinus junction (TSSJ) to the inion on both sides. This distance was then divided into three equidistant points on the left and right sides. The midpoint, denoted as the 1/2 point, corresponds to the middle of the distance between the junction point and the inion. The 1/4 point is one-fourth of the way from the inion, while the 3/4 point is three-fourths of the way from the inion in the anterior direction, respectively. All measurements are shown in millimeters (mm). Image Credits: Jonas Jurgaitis

The Frankfurt horizontal plane extending to the inion was divided into six equidistant points, encompassing the first quarter of the segment from the origin of the TSSJ junction to the inion, the middle, and the third quarter (Figure 2). The course of TS in relationship to these points was subsequently determined. The drilling was done perpendicularly to the cranial surface.

A standardized digital caliper with a precision of less than 0.01 mm was used to carry out all the measurements mentioned in the study, which were repeated three times. The statistical significance of the observed variations between the two sides was assessed using the Mann-Whitney U (Wilcoxon) test, and a significance level of p-value < 0.05 was employed to determine if the difference was statistically significant. The final outcomes were presented with two decimal points, and all measurements were recorded and reported in millimeter units.

## Results

In our study, the inferomedial point of the TSSJ was located at a median distance of 6.6 mm (3.7; 9.4) dorsally and 19.2 mm (16.1; 23.2) cranially from the digastric point in the coordinate system. The results of the TSSJ measurements are summarized in Table 1.

**Table 1:** Summary of the inferomedial point measurements (mm). The statistical analysis was conducted using the Mann-Whitney U (Wilcoxon) test.

	X on the right side, median (Q1; Q3)	X on the left side, median (Q1; Q3)	p-value	X on both sides, median (Q1; Q3)	Y on the right side, median (Q1; Q3)	Y on the left side, median (Q1; Q3)	p-value	Y on both sides, median (Q1; Q3)
Inferomedial point	6.3 (3.6; 9.2)	8.0 (4.1; 9.0)	0.695	6.6 (3.7; 9.4)	20.5 (17.4; 23.3)	18.0 (15.6; 20.6)	0.160	19.2 (16.1; 23.2)

The center of the confluence of sinuses was found to be at a median distance of 7.8 mm (4.5; 8.3) cranially to the inion, while the inferior point of the confluence of sinuses was at a median distance of 1.5 mm (0.1; 3.0) cranially to the inion. In all donated bodies studied (n = 10), the center of the confluens sinuum was 4.7 mm (3.3; 5.6) to the right in relation to the inion.

The diameter of the right transverse sinus was found to be significantly larger than that of the left transverse sinus, with a median diameter of 9.3 mm (8.6; 10.5) for the right transverse sinus, compared to a diameter of 7.0 mm (6.3; 7.8) for the left transverse sinus, with a statistical significance of p-value = 0.048.

**Figure 3:**
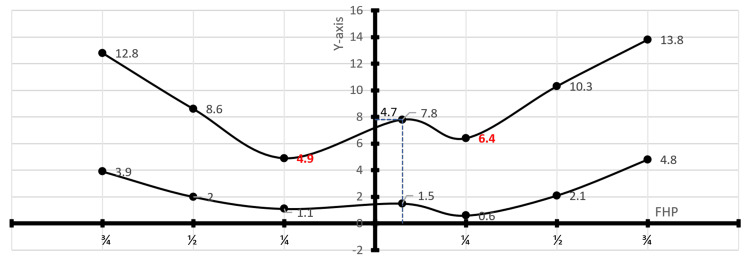
Summary of the transverse sinus measurements at six craniometric points (mm). The graph illustrates a summary of measurements of the transverse sinus at six craniometric points, presenting median values. The Y-axis denotes the line passing through the inion that is perpendicular to the Frankfurt horizontal plane (FHP). In all bodies (n = 10), the confluens sinuum center was positioned at a median value of 4.7 mm to the right relative to the inion.

The TS exiting the confluence of sinuses moves rostrally in a cranial direction with respect to the Frankfurt line. The upper boundary of the TS was found at distances of 6.4 mm (5.7; 12.7), 10.3 mm (8.8; 12.3), and 13.8 mm (11.9; 16.3) on the right side, and 4.9 mm (4.1; 7.9), 8.6 mm (7.6; 13.0), and 12.8 mm (11.7; 17.5) on the left side from the Frankfurt horizontal plane at the ¼, ½, and ¾ line points, respectively. The lower boundary was situated at distances of 0.6 mm (-2.7; 2.0), 2.1 mm (-0.8; 3.8), and 4.8 mm (2.4; 6.7) on the right side, and 1.1 mm (-3.4; 2.4), 2.0 mm (0.2; 4.8), and 3.9 mm (3.7; 5.3) on the left side from these respective points (Table 2 and Figure 3). The upper edge of the TS in relation to the Frankfurt line on the right side was significantly farther away than that on the left side (p-value = 0.027).

**Table 2:** Summary of the transverse sinus measurements at six craniometric points (mm). The statistical analysis was conducted using the Mann-Whitney U (Wilcoxon) test. ^1 ^There was a statistically significant difference in the median distance of the upper edge of the transverse sinus (TS) in relation to the Frankfurt line when comparing the right side to the left side (p < 0.05).

	Median value on the left side (Q1; Q3)	Median value on the right side (Q1; Q3)	p-value
The upper edge of the TS in relation to ¼	4.9 (4.1; 7.9)	6.4 (5.7; 12.7)	0.027^1^
The lower edge of the TS in relation to ¼	1.1 (-3.4; 2.4)	0.6 (-2.7; 2.0)	0.846
The upper edge of the TS in relation to ½	8.6 (7.6; 13.0)	10.3 (8.8; 12.3)	0.770
The lower edge of the TS in relation to ½	2.0 (0.2; 4.8)	2.1 (-0.8; 3.8)	0.375
The upper edge of the TS in relation to ¾	12.8 (11.7; 17.5)	13.8 (11.9; 16.3)	0.625
The lower edge of the TS in relation to ¾	3.9 (3.7; 5.3)	4.8 (2.4; 6.7)	0.953

## Discussion

In recent years, neurosurgeons have developed novel techniques to expose the transverse and sigmoid sinuses during craniotomy without creating significant bony defects. A critical step in this process involves precisely locating the keyhole at the TSSJ. Proper burr hole placement is crucial for the "keyhole" concept in neurosurgery, which emphasizes optimal visualization of relevant anatomical and pathological structures. Therefore, it is important to identify superficial landmarks to locate deeper structures when planning surgical approaches in the posterior fossa [12].

In the retrosigmoid craniotomy, the surgeon intends to place the initial burr hole near the transverse and sigmoid sinuses. This approach helps minimize the size of the craniotomy, reduces unnecessary exposure, and aids in early orientation. Regardless of the specific method used to uncover the sinus edge, it is essential to be able to anticipate the location of the TSSJ based on the bony landmarks of the skull. There are numerous techniques to predict the keyhole position for the retrosigmoid craniotomy. Table 3 displays a summary of the original techniques. Although there have been descriptions of at least 10 different landmarks for identifying and protecting the TSSJ, none of them is completely accurate. According to a study by Hall et al., the methods proposed by Li, Ribas, Tubbs, Teranishi, and Day are identified as the most accurate for estimating the TSSJ, as compared to other examined methods [4].

**Table 3:** Predicted keyhole position for the retrosigmoid craniotomy from external skull bony landmarks.

Author	Position
Jian ZH et al. (2022) [5]	12 mm above the top point of the mastoid groove vertically based on the Frankfurt horizontal plane
Kubo M. et al. (2021) [13]	20 mm from the posterior edge of the digastric groove, subjacent to the line extending from the digastric groove as the groove line
Teranishi Y et al. (2014) [11]	6.5 mm inferior and 6.5 mm lateral to the asterion
Tubbs RS et al. (2009) [14]	5 mm inferior to the "zygomatic line" (horizontal line parallel to the superior border of the zygomatic arch) and 10 mm posterior to the “mastoid line” (the line connecting the mastoid notch superiorly to the squamosal suture)
Ribas et al. (2005) [15]	1 cm anterior to the asterion across the parietomastoid suture
Lang and Samii (1991) [16]	30 mm behind the suprameatal spine on the Frankfurter horizontal plane

Ideally, the keyhole should provide visibility of half of the TSSJ [11]. In our study, we observed that the distance from the TMN to the IM point was found to be 19.2 mm (16.1; 23.2) cranially. Jian et al. suggested that the exact location of the burr hole should be 12 mm directly above the highest point of the mastoid groove [5]. So, considering that the burr hole cover has a diameter of 14 mm, the upper boundary of the burr hole would be approximately 0.2 cm away from the IM point of the TSSJ according to our study. In Jian et al.'s study, the distances from the keypoint to the TMN were 16.80 mm ± 0.61 (left) and 14.83 mm ± 5.13 (right). Their keypoint corresponds to our inferomedial point, which we observed to be 20.5 mm (17.4; 23.3) on the right side and 18.0 mm (15.6; 20.6) on the left side from the TMN.

In a study conducted by Ruichun Li et al. using 43 dried adult skull samples, the AS point was found to have a Y-axis value of 27.01 mm, while the IM point had a Y-axis value of 15.95 mm. Additionally, the X-axis value of the AS point was 1.92 mm anterior and the IM point was 5.46 posterior to the TMN, respectively. No significant difference was found in the sex or skull side of the AS point. However, they found statistically significant differences in the IM point between male and female skulls. The distance from the IM point to the TMN in males was shorter than that in females (14.40 vs. 19.70 mm, respectively) [10].

In our study, we utilized a Frankfurt horizontal plane to inion to delineate the trajectory of the TS after its exit from the confluence of sinuses. We found that, at the median position, the transverse sinuses were positioned above the Frankfurt line, as it exits the confluence of sinuses. The TS, exiting the confluence of sinuses, moves rostrally in a cranial direction with respect to the Frankfurt line.

According to Tubbs et al.'s study, the course of TS is most reliably recognized using a line drawn between the zygomatic arch and the inion, with 86% of transverse sinuses positioned below this line [14]. The findings of Hall et al. suggest that the asterion is a more reliable marker for identifying the lateral transverse sinus location rather than using it as a landmark for placing the initial burr hole in a retrosigmoid craniotomy [4]. Several other studies have shown that the superior nuchal line and inion are not reliable indicators for accurately locating the TS, whereas the semispinalis capitis is a more dependable anatomical structure [17-19]. It can be used for identifying the lower boundary of the medial segment of the TS during surgery. To ensure safe placement of the first burr hole in the midline infratentorial supracerebellar approach, it is recommended to position it approximately 1 cm below the inferior nuchal line, considering that the width of the proximal transverse sinus is typically 6 mm [20].

In our study, we found that the median diameter of the TS at the point of exiting the confluence of sinuses was 9.3 mm on the right side and 7.0 mm on the left side, with a statistically significant p-value of 0.048. In Bansal et al.'s study with 60 adult human dry skulls, the mean width of TS measured at the posterior, middle, and anterior parts was 10 mm ± 2.25, 9.09 mm ± 1.68, and 11.06 mm ± 1.49, respectively. Additionally, the width of the TS groove was greater on the right side, and the differences were statistically significant [21].

It is important to note that the findings of this research are applicable exclusively to adult individuals. Another limitation of this study is the possible variability among individuals in the correlation between surface landmarks and sinuses, leading to an elevated risk of sinus injury. It is important to note that our study utilized cadaveric bodies and the real clinical scenarios may differ. Prior to implementing our method in clinical practice, further validation is necessary.

## Conclusions

The TMN serves as a valuable anthropometric reference point for identifying the junction point of the transverse-sigmoid sinus. By utilizing the Frankfurt line, the path of the transverse sinus can be projected once it departs from the confluens sinuum.
